# E-perceptions and Business ‘Mating’: The Communication Effects of the Relative Width of Males’ Faces in Business Portraits

**DOI:** 10.3389/fpsyg.2021.605926

**Published:** 2021-04-16

**Authors:** Eveline van Zeeland, Jörg Henseler

**Affiliations:** ^1^Department of Design, Production & Management, University of Twente, Enschede, Netherlands; ^2^Faculty of Business and Communication, HAN University of Applied Sciences, Nijmegen, Netherlands; ^3^NOVA Information Management School, Universidade NOVA de Lisboa, Lisbon, Portugal; ^4^Department of Business Administration and Marketing, University of Seville, Seville, Spain

**Keywords:** facial metrics, non-verbal cues, online impression management, conjoint analysis, business mating

## Abstract

This study investigates the relative impacts of the facial width-to-height ratio (fWHR) on the first impressions business professionals form of business consultants when seeing their photographs on a corporate website or LinkedIn page. By applying conjoint analysis on field experiment data (*n* = 381), we find that in a zero-acquaintance situation business professionals prefer low-fWHR business consultants. This implies that they prefer a face that communicates trustworthiness to one that communicates success. Further, we have investigated the words that business professionals use to describe their preferred consultant. These approach motivations help practitioners to improve the picture-text alignment. The results underline the necessity to critically assess the pictures and text used on websites and media platforms such as LinkedIn for business purposes, and to see them as a key element of business and self-communication that can be altered in order to improve business ‘mating.’

## Introduction

“Use a picture. It’s worth a thousand words.”

–Arthur Brisbane

An ever-increasing part of our lives takes place online. Almost every business professional has a personal digital twin that is used to build online relationships and that operates not only in the private domain, but also in the business domain. In the business domain, this digital representation of a professional is visible on corporate websites and on professional social media platforms such as LinkedIn, which is currently the largest social media platform for professional purposes. In an era of personalization, everything is made personal, and these personal profiles of business professionals are more and more prominently present. However, in practice, these profiles are seldom first tested for their effects on perceivers, which is remarkable considering the fact that we “*live in a society saturated with photographic images.”* (Guthey and Jackson, 2005, p. 1057).

The professional profiles we present on corporate websites and LinkedIn not only contain facts about a business professional, they are also full of cues that people use to make inferences about the other (Pollach and Kerbler, 2011; van de Ven et al., 2017). The interpersonal perceptions of these online social cues is called *e-perception*, a term first used by Vazire and Gosling (2004). We are consciously aware of some of these verbal and non-verbal cues and manipulate them in such a way that the other will probably form a good impression of one, but other cues are more implicit.

The thing about profiles on corporate websites and social media platforms is that business professionals can control these cues; i.e., they are not influenced by a journalist or editor, as is the case for many other media (White and Raman, 1999; Murphy and Sashi, 2018). Thus, professionals can use images with positive values and can avoid ones with negative values, which in most cases is the essence of impression management (Gardner and Martinko, 1988; Parhankangas and Ehrlich, 2014). The online social environment is considered to be an excellent environment for impression management, since here the actors can completely shape the ways they self-present, from profile picture to favorite quote (Stopfer et al., 2014). Thus, they can “*manage their self-presentations more strategically than in face-to-face situations.”* (Krämer and Winter, 2008, p. 106).

### Impression Management and First Impressions

There are many ways to impress a professional other. Impression management via text by business professionals occurs at different levels: the content level, i.e., the ‘what,’ and the style level, i.e., the ‘how.’ Regarding the ‘what,’ the impression of the text can, for example, be altered by mentioning awards and nominations (Pollach and Kerbler, 2011). As to the language style, the impression of the text can be managed by the tone of voice, such as the use of positive language (Parhankangas and Ehrlich, 2014), or by the use of textual symbols, such as capitalization and emoticons (Byron and Baldridge, 2007). But more dominant than text is impression management via visual stimuli. It is in our biological nature to first look at people when forming an impression of them (Barry et al., 2015; van Zeeland and Henseler, 2018a). Faces present a large collection of informative social cues that are used to determine the emotional state, health, traits, and behavioral intentions of the face’s owner (Hess et al., 2005; Todorov et al., 2005; Rule and Ambady, 2008; Seidel et al., 2010; Re and Rule, 2015; Stephen et al., 2017). Our biological tendency to look at people’s faces occurs both offline and online (Stecher and Counts, 2008; Hum et al., 2011). From the few studies on online profile pictures generally (i.e., not specifically regarding a business context) we know that profile pictures help visitors to make judgments about an individual’s personality (Stopfer et al., 2014; Sutherland et al., 2015) and that profile owners appear to be aware of their profile pictures’ communication effects since they present pictures that are inactive, posed, appropriate, and contain only the profile owner (Hum et al., 2011). Because of that, customers can even be segmented based on their profile pictures (Vilnai-Yavetz and Tifferet, 2015). The way people pose on their profile pictures affects the impression others form. For example, individuals are perceived as being more friendly, sensitive, open and reliable when they present a profile picture in which they smile and look healthy (Turner and Hunt, 2014; Sutherland et al., 2015). Something else that might help to manage the impression in a positive way is by revealing more facial features, such that the profile picture contains more information about an individual (Steele et al., 2009). Selfies appear to have a negative impact on the impression of others: people are perceived to be less trustworthy, less socially attractive, less open to new experiences, more narcissistic and more extroverted when they take the picture themselves as compared to pictures taken by another person (Krämer et al., 2017). So, a face’s physical appearance matters concerning e-perception and a picture does appear to be “worth a thousand words,” yet we don’t know which kinds of words a face is in fact communicating.

First impressions of the other’s face have, whether we like it or not, an impact on our behaviors (Naylor, 2007; Talamas et al., 2016). Informational cues in the face activate behavioral tendencies in a viewer, and these behavioral tendencies can be classified either as approach-motivated or avoidance-motivated (Seidel et al., 2010). The behavioral tendencies of approaching and avoiding were broadly introduced by Gray (1982), and the accompanying BIS/BAS theory is a dominant theory in biological psychology (De Pascalis et al., 2013). There is one facial cue that may provoke *both* approach-motivated and avoidance-motivated behaviors: relative face width, a variable that, in the case of males, is accompanied by both socially desirable and socially undesirable correlates (Wong et al., 2011; Haselhuhn and Wong, 2012), on which we will elaborate in the next section. A relatively wide male face is said to communicate *success*, and a relatively small face is said to communicate *trustworthiness*. It is not yet known whether, concerning business portraits of business professionals on corporate websites and professional social media platforms, a relatively wide male face evokes approach-motivated behaviors from a perceiving business professional because of the *successful face* interpretation or evokes avoidance-motivated behavior because of the *untrustworthy face* interpretation. In other words: *Do business professionals prefer the successful wide face or the trustworthy small face when seeing other business professionals with whom one can potentially start a business relationship?* This is our central research question.

### E-perceptions in the Context of Business ‘Mating’

In the context of business impression management, it is relevant that cues evoke approach-motivated behaviors. How else can business relationships emerge from online profiles? While online impression management generally has attracted much attention, online impression management in a business context has been left behind, despite its strong economic relevance. Since our economy is turning into a platform-based network economy, the emergence of business relationships is becoming increasingly important for the survival and growth of businesses. In a world in which being connected appears to be the central theme, we need to know more about the emergence of interpersonal business connections.

Business relationships are often compared to romantic relationships (Dwyer et al., 1987; Perrien and Ricard, 1995; Johnston and Hausman, 2006), yet concerning business relationships we know more about the *marriage* than about the *first eye contact*. *Liking* comes before *loving*, and this process of business ‘mating’ is described as a process in which “*the characteristics of firms forming relations are not randomly matched but result from a process of assortative mating.”* (Wilkinson et al., 2005, p. 677), and is therefore comparable to romantic mating. As with romantic mating, business partners should first be aware of one another, and for this awareness, one must be attractive enough to draw the other’s attention (Dwyer et al., 1987; Mortensen, 2012). However, in the context of business mating, we don’t know in detail what attracts the other, and there is a hiatus concerning the elements that attract the other in an online environment. We seek to contribute to this knowledge from the perspective of communication psychology. This knowledge is not only of academic relevance, but is predominantly of practical relevance, because it advances professionals in their ways of being an attractive business partner (Murphy and Sashi, 2018).

We investigate the communication effects of consultants’ faces by showing *de facto* photographic images of the corporate website of a large consultancy to potential buyers of consultancy services. In this study we present only male consultants’ faces to potential buyers since the here presumed relationship between facial width and behavioral traits was, so far, only convincingly established for males. Using conjoint analysis, we investigate the preferences for a wide successful face or a small trustworthy face, so as to see whether the face width of a business consultant on a photograph on a corporate website has any effect on the choice of business professionals and whether this effect, if it exists, differs for short-term vs. long-term projects. We also study what the communication effect of the relative size of males’ face width is compared to other facial characteristics, such as attractiveness. Conjoint analysis allows us to measure the relative impact of different facial characteristics on the preference of potential buyers. Furthermore, we investigate if there is a difference between male and female professionals in the e-perceptions of the face width of a male business consultant. With this knowledge, we seek to contribute to the optimization of practitioners’ communication strategies by addressing elements that may have a strong impact, yet are easily overlooked. To strengthen this practical relevance, we also study the verbal typologies business professionals use to describe their approach motivations so that professionals have guidelines to increase picture-text alignment. We have used the HEXACO dimensions of personality to structure these verbal typologies and to facilitate the bridge from picture to text.

## The Communication Effects of the Facial Width-To-Height Ratio

**FIGURE 1:**
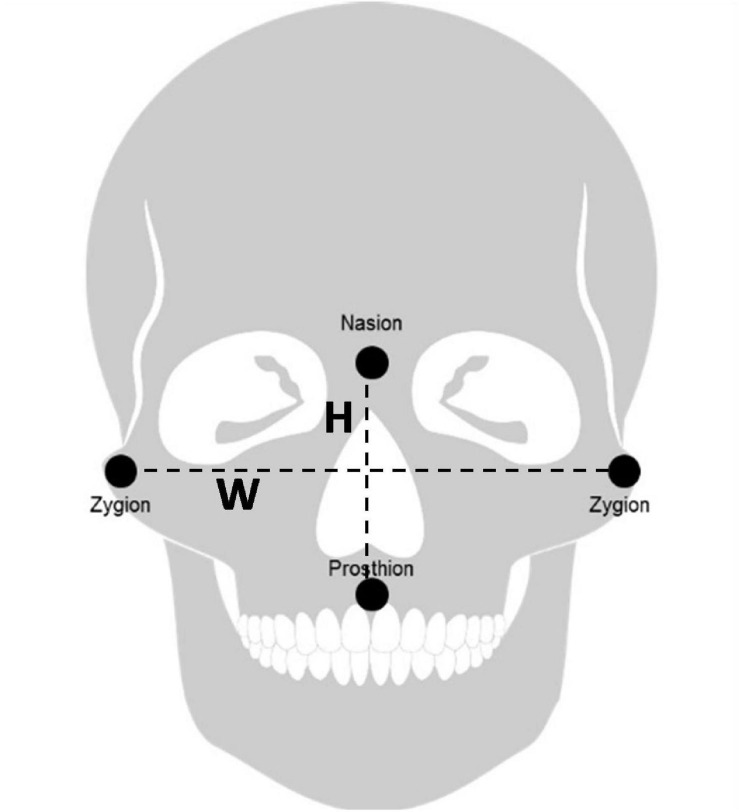
Facial width (W) versus facial height (H).

One of the cues people use when evaluating someone’s personality from their facial appearance is the facial Width-to-Height Ratio (fWHR), which is a persistent facial characteristic (i.e., it cannot be easily altered). fWHR is measured by dividing the distance between the left and right zygion (the width) by the distance between the nasion (the brow) and the prosthion (the upper lip) (the height) (see Figure 1). For males, this ratio is typically between 1.5 and 2.5. Some theorists regard fWHR as a sexually dimorphic trait, i.e., males and females exhibit different facial structure characteristics (Weston et al., 2007). Men’s fWHR is typically larger than that of women, which is hypothesized to make men seem physically more imposing (Stirrat and Perrett, 2010; Wong et al., 2011). A clear relationship between fWHR and behavior has been demonstrated only for males (Carré and McCormick, 2008; Stirrat and Perrett, 2010; Haselhuhn and Wong, 2012). Although the link between female fWHR and behavioral traits is studied by a few scholars as well, specifically with respect to sexual related behaviors, the evidence for the existence of this link is weak at the moment (Geniole et al., 2015; Arnocky et al., 2018; Zhang et al., 2018). Since our hypotheses, see the end of this literature section, rest on the assumption that a relatively wide face communicates success and a relatively small face communicates trust, and this assumption is not established for females, we therefore focus only at male fWHR and its links to behavioral traits. One of the explanations of the relationship between male facial appearance and behavior is that they share a biological cause (Zebrowitz and Collins, 1997; Re and Rule, 2015). For fWHR and the perceived accompanied behavior, it is suggested that testosterone may be this common biological cause (Lefevre et al., 2013; Alrajih and Ward, 2014; Mogilski and Welling, 2017).

### The Socially Desirable and Undesirable Correlations of a High fWHR

**FIGURE 2:**
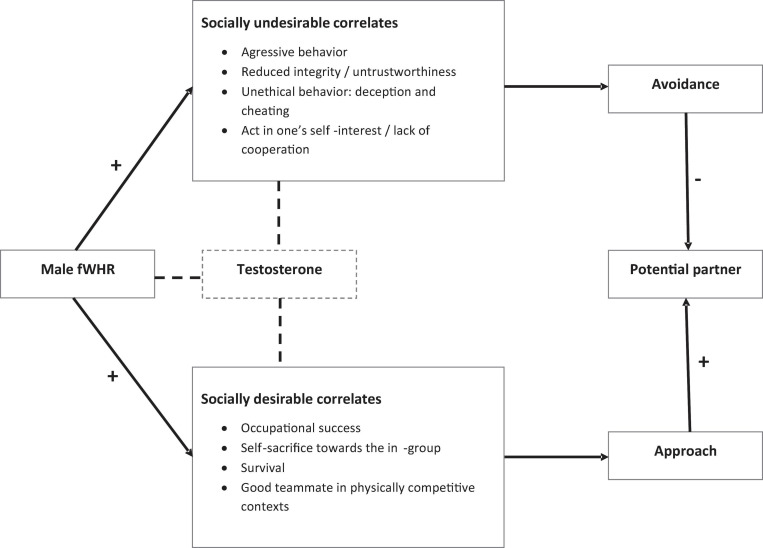
Overview of the socially desirable and undesirable correlates of high-fWHR males.

A high fWHR is accompanied by both socially desirable and undesirable correlations (Wong et al., 2011; Haselhuhn and Wong, 2012), which is visualized in Figure 2. For instance, Welker et al. (2015) found that fWHR is both positively related to the fouls committed (socially undesirable) and to goals scored (socially desirable) by football players in the 2010 World Cup. To start with the socially desirable correlates, a high male fWHR has been connected to occupational success in many ways (Little and Roberts, 2012): financial performance, leadership performance, sports, even literature. Many studies have found a positive relationship between the fWHR of a man in charge and his financial and leadership performance (Rule and Ambady, 2008; Wong et al., 2011; Re and Rule, 2016). “*Thus, some element of financial success seems to be communicated through facial appearance”* (Rule and Ambady, 2008, p. 110). One explanation for this finding is that males with a higher fWHR have a stronger sense of power, which is associated with the tendency to view the environment more optimistically, to see more opportunities, and to focus on the bigger picture (Wong et al., 2011; Haselhuhn and Wong, 2012). fWHR has been related to success in sports (Welker et al., 2015). For instance, Tsujimura and Banissy (2013) showed a correlation between the fWHR of professional Japanese baseball players and their home run performance. High-fWHR authors even tend to be nominated for the Nobel Prize in literature at a younger age (Lebuda and Karwowski, 2016). Males with a higher fWHR may have more success, because they strive for success more (Lewis et al., 2012). But higher-fWHR males also appear to be able to sacrifice themselves to benefit the in-group (Stirrat and Perrett, 2012). Males’ fWHR is even linked to their survival: low-fWHR males are more likely to die from contact violence (Stirrat et al., 2012). High-fWHR males are considered to be good teammates in physically competitive environments (Hehman et al., 2015). In other words, you want these guys on your team when you compete against other teams. However, these are also the guys one avoids if the environmental condition is such that cooperation instead of competition is needed. High-fWHR males are superior negotiators in competitive bargaining, but are less likely to reach an agreement in an exercise that requires cooperation (Haselhuhn et al., 2014). People show more caution when interacting with a high-fWHR male (Haselhuhn et al., 2013), because there is a dark side to fWHR: a high fWHR also has some socially undesirable correlations. First, male fWHR has been correlated to aggressiveness (Carré and McCormick, 2008; Carré et al., 2009; Lefevre and Lewis, 2014; Haselhuhn et al., 2015; Welker et al., 2015). The fWHR-aggressiveness relationship appears to be moderated by social status: only in the context of a low social status does variability in fWHR predict individual differences in aggressive behaviors (Goetz et al., 2013). However, Gómez-Valdés et al. (2013) noted that there is insufficient support to state that males with wider faces are more aggressive, yet they are at least perceived as more dominant and intimidating (Hehman et al., 2013b; Alrajih and Ward, 2014; Valentine et al., 2014) and feel more powerful (Haselhuhn and Wong, 2012). High-fWHR males are also perceived to have less integrity and to be more untrustworthy (Stirrat and Perrett, 2010; Ormiston et al., 2017). fWHR has also been brought in relation to prejudicial beliefs (Hehman et al., 2013a) and to unethical behaviors, such as deception and cheating (Haselhuhn and Wong, 2012). Geniole et al. (2014) found that the fWHR-cheating relationship is mediated by the psychopathic personality factor of fearless dominance. High-fWHR males tend to act in self-interest and show a lack of cooperation (Haselhuhn and Wong, 2012; Haselhuhn et al., 2013). So, it is not without reason that organizations choose CEOs with narrow faces in the context of leadership replacement after financial misconduct; they want new faces that communicate integrity (Gomulya et al., 2017).

### Hypotheses

Interestingly, fWHR is both correlated to attitudes and behaviors that one likes to approach and to attitudes and behaviors one likes to avoid. In other words, a high-fWHR male seems to simultaneously attract and reject the other. In the context of romantic mating, it has been observed that women want to approach high-fWHR males for short-term relationships but not for long-term ones (Valentine et al., 2014). We explore how these effects are displayed in a professional business context. In a professional business context, business people can seek mates in the form of a consultant. Consultants are hired in the short term, mostly to deal with a hiatus in knowledge or expertise (the expert model). But relationships with consultants can also be long term, for instance when a consultant is a firm’s standard external strategic advisor or when they assist in projects that take multiple years (the coach model). One can expect this type of business mating to be characterized by the same preference distribution type as romantic mating (Valentine et al., 2014), leading to the following hypotheses:

H1a: The male business consultant’s fWHR affects a business professional’s preference.

If H1a is accepted:

H1b: For the short-term condition (the expert model), business professionals prefer high-fWHR faces that communicate success.

H1c: For the long-term condition (the coach model), business professionals prefer low-fWHR faces that communicate trustworthiness.

Clearly, more variables affect business partner choices based on first impressions than only fWHR. Attributes such as attractiveness (Langlois et al., 2000; Valentine et al., 2014), affective or kind expressions (Hess et al., 2005; Rule and Ambady, 2008), facial maturity or estimated age (Friedman and Zebrowitz, 1992; Zebrowitz and Montepare, 2005), and perceived intelligence (Hehman et al., 2015) are also expected to influence perceptions. We seek to measure the relative impact of fWHR, controlled for estimated age, on the selection of a consultant for a first interview purely based on a first impression as acquired from a profile photograph. Facial attractiveness is the most important factor when forming a first impression of a business consultant, as has been found concerning online profile pictures (Turner and Hunt, 2014). Being attractive has not only proven to be beneficial in the context of a romantic relationship, but also in the context of more professional relationships. Attractive people are generally considered to be more competent, which is known as the *attractiveness halo effect* (Dion et al., 1972; Ritts et al., 1992; Ahearne et al., 1999; Toledano, 2013; Re and Rule, 2015). Thus, business professionals likely prefer an attractive consultant over an unattractive one if there is zero acquaintance between the information buyer and seller, because attractiveness positively colors the general impression.

H2a: The relative impact of a business consultant’s attractiveness on a business professional’s preference is bigger than that of fWHR.

Concerning perceived intelligence, it makes sense that this is what business professionals look for when hiring consultants. One can argue that consultants deliver knowledge and intelligence, so that any cue that signals knowledge or intelligence must be important. The face is a valuable source of cues: people can accurately evaluate men’s intelligence by just looking at a photograph (Kleisner et al., 2014). Owing to intelligence’s importance in the consulting context, in which this study is positioned, we expect its impact to be stronger than that of fWHR.

H2b: The relative impact of a business consultant’s perceived intelligence on a business professional’s preference is bigger than that of fWHR.

Concerning perceived kindness, we don’t expect a strong effect. In the business literature, kindness is predominantly discussed in business ethics and is brought into relationship with Confucianism (Romar, 2002). It is always nice if people are kind, but kindness by itself in a business context is not an economically valuable characteristic.

H2c: The relative impact of a business consultant’s perceived kindness on a business professional’s preference is smaller than that of fWHR.

Finally, we expect that the gender of the business professional who is looking at the business consultants’ photographs is having an impact on the e-perceptions of the male consultants, which is affecting the preferences. Men and women process pictures differently. For instance, women show a stronger defensive and aversive reaction to unpleasant pictures (Bradley et al., 2001; Hillman et al., 2004; Stins et al., 2011). So, women place different values on the same stimuli than men.

H3: Male and female business professionals place different importance values on fWHR, attractiveness, intelligence, and kindness when looking at business consultants’ photographs.

## Materials and Methods

To measure fWHR’s *relative* impact on the selection for a first interview based on a first impression, we used a conjoint analysis. Conjoint analysis is often used in market research to determine an attribute’s relative importance. In the psychology domain conjoint analysis is less common than for example regression analysis. However, conjoint analysis “involves the measurement of psychological judgments” (Karakaya and Awasthi, 2014, p. 123) with respect to preferences. Specifically regarding the nature of our research question, i.e., human mating, conjoint analysis was used before, also by scholars from the psychology domain (Mogilski et al., 2014; Mogilski and Welling, 2017). Furthermore, conjoint analysis has been found to be more robust and accurate than multiple linear regression when the data is orthogonal and the sample size is relatively small (Karakaya and Awasthi, 2014). Conjoint analysis as implemented in SPSS relies on OLS regression (SPSS Inc, 1997; IBM, 2012). It assumes that an object can be split into different components. In this case, it assumes that a face can be split in components that can be judged separately. Since it is not uncommon to analyze faces’ components, this assumption seems valid (Zebrowitz and Montepare, 2008; Mogilski and Welling, 2017).

Since facial features appear to be correlated to behavioral traits, and these behavioral traits appear to be correlated to occupational success, a field experiment with *de facto* business consultants is the best way to deal with these correlations. For a field experiment (as opposed to a lab experiment), we needed real photographs of real business consultants, and these photographs had to be very similar in quality and positioning. We will now outline the different methodological steps of our field experiment using conjoint analysis.

### Stimuli

In our search for photographs of actual consultants, we came across a well-known Dutch consultancy that presents photos of all their consultants in exactly the same way on their company website. This consultancy has more than 200 consultants working in both the public and private sectors on a wide range of managerial and governmental issues. All its photographs are taken by the same photographer, and with the same camera, background, and arrangement (the importance of these camera parameters is made explicit in Kramer, 2016). This consultancy gave written consent to use these photographs for our study. All the individual consultants were also asked for their consent, and we used only the photographs of consultants who gave written consent. Since both the biological dimension (age, race, gender, etc.) and the environmental one (clothing, social group, etc.) have an impact on how people are judged, we wanted the stimuli to be as homogeneous as possible along these dimensions. We selected photographs of white male consultants with no potentially distracting features, such as glasses or facial hair, and who all wore the same type of suit. All the faces directed their gaze toward the viewer. Of 140 photographs of male consultants, we could not use 111, owing to distracting features or a lack of a direct gaze. Of the 29 selected photographs, all contained 354 pixels, 24 consultants gave written consent. So, we had 24 stimuli.

### Measurement of fWHR

We calculated these 24 faces’ fWHR using Image J Software. For each consultant, four members of the research team calculated the fWHR, and then we averaged the measurements to create a single fWHR score. These mean fWHRs ranged from 1.83 to 2.33, with a mean of 2.07 (Table 1 presents the mean fWHRs of the consultants selected for the conjoint analysis). When the fWHR is measured from a photograph, the exact positions of the right and left zygion, the nasion, and the prosthion are hard to determine; thus, it is common to let different trained researchers calculate fWHR (for more information on the measurement of fWHR, see Weston et al., 2007; Lefevre et al., 2013; Alrajih and Ward, 2014).

**TABLE 1:** Orthogonal array and preferences by business professionals.

Card ID	fWHR	Perceived unattractiveness	perceived kindness	PREF 1 ST	PREF 2 ST	PREF 9 ST	PREF 10 ST	PREF 1 LT	PREF 2 LT	PREF 9 LT	PREF 10 LT
1	**High** (*M* = 2.15, *SD* = 0.06)	**Unattractive** (*M* = 33, *SD* = 16), (NA = 90, FA = 10, VA = 0)	**Very kind** (*M* = 72, *SD* = 16), (NK = 2, FK = 53, VK = 45)	6.8	5.5	14.2	13.1	5.0	5.8	14.2	11.3
2	**High** (*M* = 2.10, *SD* = 0.06)	**Not unattractive** (*M* = 53, *SD* = 18), (NA = 21, FA = 68, VA = 11)	**Kind** (*M* = 69, *SD* = 17), (NK = 2, FK = 66, VK = 32)	9.7	11.8	8.4	10.2	8.4	9.2	9.7	14.7
3	**Low** (*M* = 1.93, *SD* = 0.04)	**Not unattractive** (*M* = 53, *SD* = 20), (NA = 31, FA = 55, VA = 14)	**Very kind** (*M* = 80, *SD* = 14), (NK = 0, FK = 35, VK = 65)	25.7	17.6	4.5	2.4	18.6	15.5	3.9	4.2
4	**High** (*M* = 2.29, *SD* = 0.04)	**Not unattractive** (*M* = 46, *SD* = 19), (NA = 44, FA = 53, VA = 3)	**Very kind** (*M* = 80, *SD* = 12), (NK = 0, FK = 27, VK = 73)	12.1	10.5	10.2	6.3	11.8	8.9	7.9	7.1
5	**Low** (*M* = 1.95, *SD* = 0.05)	**Unattractive** (*M* = 40, *SD* = 21), (NA = 60, FA = 35, VA = 5)	**Very kind** (*M* = 71, *SD* = 15), (NK = 3, FK = 52, VK = 45)	1.6	5.8	13.6	15.5	2.4	3.9	15.0	20.2
6	**High** (*M* = 2.18, *SD* = 0.05)	**Unattractive** (*M* = 28, *SD* = 17), (NA = 92, FA = 8, VA = 0)	**Kind** (*M* = 64, *SD* = 19), (NK = 5, FK = 63, VK = 32)	2.4	4.5	20.5	22.6	3.1	6.6	17.1	16.8
7	**Low** (*M* = 1.92, *SD* = 0.05)	**Not unattractive** (*M* = 59, *SD* = 21), (NA = 16, FA = 53, VA = 31)	**Kind** (*M* = 67, *SD* = 14), (NK = 2, FK = 79, VK = 19)	15.0	15.0	4.5	5.5	13.9	13.9	5.8	6.6
8	**Low** (*M* = 1.83, *SD* = 0.04)	**Unattractive** (*M* = 33, *SD* = 18), (NA = 86, FA = 14, VA = 0)	**Kind** (*M* = 66, *SD* = 15), (NK = 10, FK = 69, VK = 21)	5.8	6.3	10.5	7.9	9.2	9.4	11.5	6.6
9^*h*^	**High** (*M* = 2.12, *SD* = 0.05)	**Not unattractive** (*M* = 52, *SD* = 17), (NA = 32, FA = 55, VA = 13)	**Very kind** (*M* = 71, *SD* = 13), (NK = 3, FK = 53, VK = 44)	9.2	9.4	6.3	5.8	8.4	11.0	7.9	5.2
10^*h*^	**Low** (*M* = 2.02, *SD* = 0.04)	**Unattractive** (*M* = 33, *SD* = 19), (NA = 71, FA = 27, VA = 2)	**Very kind** (*M* = 75, *SD* = 18), (NK = 2, RK = 31, VK = 67)	11.8	13.6	7.3	10.8	19.2	15.7	7.1	7.3

*On the left the orthogonal array for the 2 × 2 × 2 design for the conjoint analysis (h, holdout card). The mean estimated age of the consultants on the ten selected photographs to match the cards is 39 (range 31–48). fWHR shows the mean fWHR out of four measurements. The number showed with perceived unattractiveness and kindness is the average score of 62 students on a scale from 0 to 100. Furthermore the frequencies in percentages over the three categories are presented (NA, not attractive; FA, fairly attractive; VA, very attractive; NK, not kind; FK, fairly kind; VK, very kind). On the right side of the table the frequencies in percentages of consultants selected as first or second preference or as ninth or tenth preference for both the short-term (ST) scenario in the expert-model and the long-term (LT) scenario in the coaching model are presented (*n* = 381 business professionals).*

### Measurement of Other Variables

To get rankings on estimated age, attractiveness, perceived intelligence, and perceived kindness, the 24 photographs of professional consultants were judged via a survey, for which Qualtrics Survey Software was used. The participants were students at a business school at a Dutch University of Applied Science. They were explicitly told that this survey was about their first impression, and that they had to follow their intuition. Every element was judged in two ways: an estimating way (for instance, on a scale from 0 to 100, how intelligent do you consider this consultant to be?) and a categorizing way (for instance, do you consider this consultant to be very intelligent, fairly intelligent, or not really intelligent?). By including these two measure types, we could mitigate individual differences in the perceptions of high and low rankings.

Sixty two business school students (mean age: 19.90, *SD* = 1.97, range = 16–24; 43.5% male, 56.5% female) judged 24 business consultants’ photographs along estimated age, attractiveness, perceived intelligence, and perceived kindness. Unfortunately, the results showed too little variety on the trait *perceived intelligence*: almost all consultants were perceived as fairly intelligent. Thus, we had to omit this trait for the rest of the study, because we could not distinguish different levels in the component, which is necessary for conjoint analysis. Thus, we were unable to test H2b. Concerning the trait *attractiveness*, something interesting occurred: the respondents displayed fairly negative perspectives on the consultants’ attractiveness. This may be partly caused by the age difference between the judges of the photographs and the people in the photographs. But another logical explanation for this finding is that there were no models on the pictures but actual business consultants. Thus, for the rest of the study, the trait attractiveness was reversed into unattractiveness, in which there are two levels: a consultant is either unattractive or not unattractive. Concerning the trait *perceived kindness*, the respondents were much more positive. No consultant was considered by more than one-third of the respondents as *not kind.* Thus, for the rest of the study, the trait *perceived kindness* has two levels: a consultant is either perceived as *kind* or as *very kind*. To control for estimated age, and thus for facial maturity, for the rest of the study, we used only photographs of consultants that were estimated to be between 30 and 50 years old, and excluded consultants perceived as a junior or as a senior.

### Orthogonal Design

Next, we transformed every consultant into a profile based on the rankings on the three remaining variables fWHR, (un)attractiveness, and kindness; for instance, having a high fWHR, being not unattractive, and appearing very kind. We then used the Generate Orthogonal Design procedure of SPSS to generate an orthogonal array, which is the necessary first step in conjoint analysis. The orthogonal array is a representative set of combinations of different levels for different factors, in which every combination is represented by a card or profile. Based on our 2 × 2 × 2 design, this resulted in a set of 10 cards with two holdout cards (see Table 1). Holdout cards are rated by the subject but are not part of the model; they are used to test the model. For each card in the orthogonal array, the best fit was selected: the photograph of an actual business consultant that best met the requirements. The survey respondents judged the 24 consultants’ estimated age, attractiveness, perceived intelligence, and perceived kindness twice: once on a scale from 0 to 100 (estimating) and once by choosing between three categories (categorizing). Both measures were used to compile profiles of the consultants (see left side of Table 1). When multiple photographs of consultants matched the required profile for a card, the face with the highest fWHR when a high fWHR was needed, or the lowest fWHR when a low fWHR was needed, was selected.

### Field Experiment

We then presented the photographs representing the 10 cards of the orthogonal array to business professionals who can be considered to be potential buyers of business consultants. We invited business professionals via LinkedIn to take part in the research, which is a form of convenience sampling. Convenience sampling is a non-random sampling method that is predominantly used owing to its speed, low-threshold use, and ease of accessibility by research participants (for an overview on the pros and cons of convenience sampling, see Etikan et al., 2016). This sampling method made it possible for us to reach a large sample of highly educated business professionals, which was necessary because highly educated business professionals are most likely to be actual or potential buyers of consultants’ services.

In the form of a survey, for which Qualtrics Survey Software was used, we asked the professionals to order the photographs in a way that reflected their preference for inviting the consultant for a first interview^1^. The invitation of a consultant for a first interview is an approach measurement. The order of the photographs was randomized. The respondents had to order the same photographs set twice: once for the condition in which a consultant was needed just to fill a knowledge and expertise gap on a project (short-term condition, the expert model) and once for the condition in which a consultant was needed as a coach on a long project in which the firm’s new strategy was defined and in which delicate information must be shared (long-term condition, the coach model). This within-subject design with conjoint analysis was also used by Mogilski and Welling (2017) when they investigated fWHR’s relative impact on the preference for romantic mating in a short-term vs. a long-term relationship. We used the same procedure for business mating. Since we performed a field experiment with the photographs of real consultants, one of the consultants could easily be someone’s friend, brother, or consultant. Thus, as a final control question in the survey, we asked the respondents whether they knew one of the consultants. We excluded the respondents who answered yes to this control question from further analysis.

### Categorization of Approach Motivations Using HEXACO Dimensions

For both the short-term and the long-term conditions, we also asked the respondents to describe their first choice with keywords. We categorized these verbal descriptions of the preferred consultant *ex post* using the HEXACO dimensions of personality structure (Ashton et al., 2004; Lee and Ashton, 2008), to be able to give some structured elucidation to the approach motivations. The HEXACO structure is a six-dimensional personality framework in which HEXACO is an acronym for the dimensions honesty/humility (H), emotionality (E), extraversion (X), agreeableness (A), conscientiousness (C), and openness to experience (O). In short, this is the ‘Big Five model plus one’ (Rafi et al., 2013), although the interpretations of some of the five dimensions differ in the HEXACO structure. The sixth dimension, the honesty/humility dimension, was added to the Big Five model to categorize typologies as sincere, honest, and modest. Since in the evaluation of trustworthy vs. successful faces, many descriptions will likely fall in the honesty/humility domain, the HEXACO structure is a better fit than the traditional Big Five structure. In a work context, the honesty/humility domain has already been brought in relationship to (reduced) integrity (Marcus et al., 2007), which matches the path of the socially undesirable coordinates of high fWHR. Bourdage et al. (2015) also stressed the importance of the honesty/humility dimension for the investigation of impression management behaviors. Notably, we used the HEXACO structure only as a way to categorize the qualitative data (i.e., the business professionals’ impressions of the business consultants), and not as a way to assess the consultants’ personality structures.

## Results

We present the results based on four analysis types: a descriptive analysis including the sample characteristics, the conjoint analysis, an analysis for unobserved heterogeneity, and an analysis of the qualitative approach motivations using the HEXACO structure.

### Descriptive Analysis

There were 391 complete responses to the survey. After correcting for subjects who wrote that they knew one of the consultants, who had difficulties making a preference, or who had other difficulties answering the survey, there were 381 remaining complete responses (mean age 36, range 18–70; male 42%, female 58%). Of the subjects, 90% was highly educated (45% Bachelor degree, 39% Master’s degree, 6% Ph.D.), and 44% had bought a consultancy service during their career or had worked intensively with a consultant.

Looking at the preferences for consultants in the short-term condition (the expert model) (see Table 1), the top two and bottom two preferences revealed that the two consultants with low fWHRs and fairly attractive faces (cards 3 and 7) were selected the most as top preference and the least as the bottom preference (ninth or tenth). For the long-term condition (the coach model), card 10 was the top preference, which also had a low fWHR but was considered less attractive. The consultant who stood out for not being preferred as first or second, but had a very high frequency in the bottom preference, card 6, showed an opposite profile: high fWHR and an unattractive face. Interestingly, the preferences were not completely stable: for the long-term condition, there were different frequencies regarding the preferences for consultants than for the short-term condition.

### Conjoint Analysis

The conjoint analysis results appear in Table 2. The correlations in both the short-term and the long-term conditions were acceptable. They serve only as validation, and show that the predictions based on the conjoint analysis largely correlate to actual choices people make (holdout cases). The most interesting result is that fWHR does matter; thus, H1a is accepted. In contrast to what one would expect, there was no significant difference between the results for the short-term and for the long-term conditions. So, the context and nature of consulting work does not seem to affect the factors that influence the first impressions and preferences at the micro-level. Both for the short-term and the long-term conditions, there was an inverse fWHR-preference relationship: low-fWHR consultants were preferred over high-fWHR consultants. Thus, H1b was rejected and H1c was accepted.

**TABLE 2:** Results conjoint analysis (*n* = 381) in both the short-term and the long-term condition.

		Short-term condition (expert model)			Long-term condition (coach-model)		
Factor	Level	Utility estimate	Standard error	Importance values	Utility estimate	Standard error	Importance values
fWHR	Low	–0.676	0.329	29.328	–0.602	0.427	34.228
	High	–1.352	0.658		–1.205	0.855	
Unattractiveness	Unattractive	1.440	0.329	62.472	1.156	0.427	65.697
	Not unattr.	2.879	0.658		2.312	0.855	
Kindness	Kind	0.189	0.329	8.200	–0.001	0.427	0.075
	Very kind	0.378	0.658		–0.003	0.855	
	Constant	3.071	0.870		3.671	1.131	

**Correlations**		**Value**	**Sig.**		**Value**	**Sig.**	
Pearson’s *R*		0.925	0.000		0.836	0.005	
Kendall’s tau		0.786	0.003		0.571	0.024	

Attractiveness (or being not unattractive) is considered the most important factor. On the other hand, perceived kindness did not seem to play a distinct role. So, business professionals predominantly prefer attractive, or at least not unattractive, consultants and, second, low-fWHR consultants. H2a and H2c could be accepted.

Table 3 presents the conjoint analysis results as split by gender. There was no big difference between male and female business professionals regarding the importance values attached to the facial characteristics of preferred business consultants. Both males and females appeared to prefer business consultants who are not unattractive over unattractive ones and preferred low-fWHR over high-fWHR consultants; further, for both males as females, attractiveness was more important than fWHR. For male business professionals, the attractiveness of male consultants is definitely no less important than for female professionals, in contrast to what one may expect. Perceived kindness appeared more important for females than for males, but this should be interpreted with caution, given the low importance values and the high standard errors. So, we had to reject H3.

**TABLE 3:** Results conjoint analysis by male (*n* = 160) and female (*n* = 220) participants.

		Males (short-term condition)			Females (short-term condition)		
Factor	Level	Utility estimate	Standard error	Importance values	Utility estimate	Standard error	Importance values
fWHR	Low	–0.616	0.320	32.137	–0.736	0.350	27.621
	High	–1.231	0.640		–1.473	0.701	
Unattractiveness	Unattractive	1.266	0.320	66.069	1.584	0.350	59.420
	Not unattr.	2.531	0.640		3.168	0.701	
Kindness	Kind	–0.034	0.320	1.794	0.345	0.350	12.958
	Very kind	–0.069	0.640		0.691	0.701	
	Constant	3.577	0.847		2.710	0.927	

**Correlations**		**Value**	**Sig.**		**Value**	**Sig.**	
Pearson’s *R*		0.910	0.001		0.931	0.000	
Kendall’s tau		0.786	0.003		0.857	0.001	

### Unobserved Heterogeneity

In the previous analysis, we assumed that all individuals acted along the same underlying model. However, this is not necessarily so. There may be the potential validity threat of unobserved heterogeneity (Becker et al., 2013), which means that the sample actually consists of a finite set of subsamples, all of which have their idiosyncratic mechanism. Fortunately, since utilities are determined on an individual basis, we can explore the extent to which the results may be affected by unobserved heterogeneity.

To examine for unobserved heterogeneity, we conducted a two-step cluster analysis on the individual utilities, using a log likelihood distance measure. In step 1, a Cluster Features Tree (CFT) was constructed; in step 2, the leaf nodes of the CFT were grouped. Clusters were created by the agglomerative clustering algorithm, which brings forward the best number of clusters based on the Schwarz’s Bayesian Inference Criterion (BIC). In this case, four clusters were identified.

To shed light on the idiosyncratic mechanisms working for each cluster and to see which choice behavior differences they caused, we redid the conjoint analysis for each subsample (see Table 4). Notably, this did not alter the individual utilities. The results indicate that, for some individuals, fWHR was more important than for others. Cluster 3 stands out most, not only owing to fWHR’s high importance, but also because the relationship between fWHR and the utilities is positive. These results indicate that business professionals don’t perceive consultants’ faces in the same way, and that general results cannot be generalized. When cluster 3 is discarded, total utility = –1.090 ^∗^ fWHR + 1.862 ^∗^ unattractiveness + 0.139 ^∗^ kindness, with the importance values 35.251 (fWHR), 60.238 (unattractiveness), and 4.510 (kindness).

**TABLE 4:** Results conjoint analysis by cluster (unobserved heterogeneity analysis).

		*Cluster 1 (short-term condition)*			*Cluster 2 (short-term condition)*		
Factor	Level	Utility estimate	Standard error	Importance values	Utility estimate	Standard error	Importance values
fWHR	Low	–0.447	0.385	12.851	–1.034	0.264	21.449
	High	–0.895	0.770		–2.068	0.528	
Unattractiveness	Unattractive	2.970	0.385	85.313	1.418	0.264	29.403
	Not unattr.	5.940	0.770		2.836	0.528	
Kindness	Kind	–0.064	0.385	1.836	2.370	0.264	49.148
	Very kind	–0.128	0.770		4.740	0.528	
	Constant	0.812	1.019		0.370	0.699	

**Correlations**		**Value**	**Sig.**		**Value**	**Sig.**	
Pearson’s R		0.969	0.000		0.984	0.000	
Kendall’s tau		0.786	0.003		0.929	0.001	

		***Cluster 3 (short-term condition)***			***Cluster 4 (short-term condition)***		
**Factor**	**Level**	**Utility estimate**	**Standard error**	**Importance values**	**Utility estimate**	**Standard error**	**Importance values**

fWHR	Low	1.196	0.491	57.491	–1.934	0.468	50.184
	High	2.391	0.982		–3.868	0.936	
Unattractiveness	Unattractive	–0.471	0.491	22.648	0.778	0.468	20.196
	Not unattr.	–0.942	0.982		1.557	0.936	
Kindness	Kind	0.413	0.491	19.861	–1.142	0.468	29.621
	Very kind	0.826	0.982		–2.283	0.936	
	Constant	2.793	1.299		7.946	1.239	

**Correlations**		**Value**	**Sig.**		**Value**	**Sig.**	
Pearson’s *R*		0.809	0.008		0.930	0.000	
Kendall’s tau		0.714	0.007		0.786	0.003	

### Analysis of the Qualitative Descriptions: The Approach Motivations

We asked all the respondents to describe their first impressions of their preferred consultant in a few words. We scored and categorized these qualitative descriptions of the first preference *ex post* using the HEXACO personality structure dimensions (see Table 5). First, these results show business professionals’ approach motivations: the words business professionals use to describe the potential business partner they would want to approach. Words in the honesty/humility domain were used the most. This was an expected outcome and the reason why we used the HEXACO model over the Big Five model to structure the qualitative data (see section “Categorization of Approach Motivations Using HEXACO Dimensions”).

**TABLE 5:** Approach motivations: relative distribution of qualitative descriptions, categorized by using the HEXACO-model of personality structure.

Dimension	Terms	Total set	Cluster 1, 2 and 4	Cluster 3	Two-tailed
					
		381 individuals 1118 descriptions	312 individuals 926 descriptions	69 individuals 192 descriptions	*p*-value
Physical Appearance	Experienced, seniority, young professional, well-groomed, informal, formal, smile, eyes, pleasant face	13.15%	14.25%	7.81%	0.016
H: honesty/humility	Trustworthy, kind, friendly, honest, down-to-earth, normal, sympathetic, modest, nice, warm, benevolent, helping, thinking along, empathically, loyal, polite	34.44%	33.15%	40.63%	0.047
E: emotionality	Balanced, stable, persuasive, strong, persistent	0.98%	1.08%	0.52%	0.475
X: extraversion	Social, open, approachable, humor, energetic, dynamic, fresh, optimism, positive, cheerful, enthusiasm, spontaneous, (self-)confident, communicative, (pro-)active, naughty	22.81%	23.00%	21.88%	0.735
A: agreeableness	Calm, patient, good listener, interested, flexible, relaxed, equal, safe, team player	4.65%	4.64%	4.69%	0.979
C: conscientiousness	Serious, businesslike, professional, pragmatic, realistic, entrepreneurial, ambitious, reliable, diligent, directly, structured, efficient, well-prepared, responsible, goal-oriented	6.98%	6.91%	7.29%	0.851
O: openness to experience	Intelligent, wise, capable, expertise, open-minded, creative, innovative, modern, up-to-date, critical, curious	16.99%	16.95%	17.19%	0.938

*The right column presents the two-tailed *p*-value to assess if the frequencies for cluster 3 are significantly different from the frequencies for cluster 1, 2, and 4.*

The dominant approach motivations were trustworthy (14.85%), intelligent or wise (10.47%), kind or friendly (10.29%), and social, open, or approachable (9.03%). Trustworthiness’ dominance is in line with the results from the conjoint analysis, showing the preference for a *trustworthy* face (low fWHR) over a *successful* one (high fWHR). *Intelligent* or *wise* were the words used second most often. Considering the task, i.e., which consultant would you invite for a first interview if you needed someone to fill a knowledge gap, it is perhaps surprising that *intelligent* was not used more often by business professionals to describe their preferred consultant.

Because the cluster analysis revealed that one cluster has a positive relationship between fWHR and preference, in contrast to the other groups, we compared the qualitative descriptions of cluster 3 to the other clusters. Of cluster 3’s members, 72.3% chose a high-fWHR consultant as their first preference, compared to 33.1% of the other clusters’ members, which is a significant difference (*p* ≤ 0.001). Yet, remarkably, cluster 3’s members did not really use other words to describe their first preference, and where they did, they used words in the *honesty* domain more. This finding is somewhat surprising, given that they preferred a *successful* face over a *trustworthy* one. This gives us reason to assume that these qualitative descriptions are *ex post* rationalizations, i.e., something implicit is brought to the explicit level.

## Discussion and Conclusion

We have focused on a micro-foundation of choice behaviors in business mating: the possible influence of the relative face width of a business consultant on business professionals’ choice behaviors. The most striking result is that fWHR does in fact matter for business professionals’ preferences for a business consultant, and thus that relative face width does in fact have a communication effect. It is not the successful face—a high-fWHR face that is preferred the most by most business professionals, but it is the trustworthy face—the low-fWHR face. This finding holds for both short-term and long-term consulting projects. Being not unattractive is more important for the chance of being preferred than fWHR. Perceived kindness does not matter, and can be seen as a luxury facial trait: nice if it’s there, but it doesn’t really matter if it’s not (Li et al., 2002). Table 6 presents an overview over the hypotheses and the test outcomes. Notably, we have identified unobserved heterogeneity in the data, so there may be groups of business professionals who reacted differently to others to the business consultants’ faces in a zero-acquaintance situation.

**TABLE 6:** Overview of the tested hypotheses.

Hypothesis		Accept/Reject	Clarification
H1a	The male business consultant’s fWHR affects a business professional’s preference.	*Accepted*	
H1b	For the short-term condition (the expert model), business professionals prefer high-fWHR faces that communicate success.	*Rejected*	In the short-term condition business professionals prefer low-fWHR consultants, just as in the long-term condition.
H1c	For the long-term condition (the coach model), business professionals prefer low-fWHR faces that communicate trustworthiness.	*Accepted*	
H2a	The relative impact of a business consultant’s attractiveness on a business professional’s preference is bigger than that of fWHR.	*Accepted*	
H2b	The relative impact of a business consultant’s perceived intelligence on a business professional’s preference is bigger than that of fWHR.	*Unable to test*	There was too little variation in the perceived intelligence of the faces of the consultants, so the trait intelligence could not be included in the conjoint analysis.
H2c	The relative impact of a business consultant’s perceived kindness on a business professional’s preference is smaller than that of fWHR.	*Accepted*	Note: the impact of perceived kindness is very small and does not play any substantial role in decision making.
H3	Male and female business professionals place different importance values on fWHR, attractiveness, intelligence, and kindness when looking at business consultants’ photographs.	*Rejected*	Although some differences between male and female respondents are visible, the differences are too small to be able to accept this hypothesis.

Based on the study results, it can be suggested that the impression of trustworthiness is a necessary condition for a relationship to develop. First impression processes are very likely important for the assessment whether or not to avoid the other, since negative impressions are formed swiftly and are more salient than positive ones (Baumeister et al., 2001; Gomulya et al., 2017), and since the question whether or not to go into business with each other is answered in a later phase (van Zeeland and Henseler, 2018b). This may explain why, in the starting phase of a potential business relationship in a zero-acquaintance situation, business professionals prefer a *trustworthy* face over a *successful* one. So, one of the first things we look for in a business context is cues that tell us whether or not the other may cheat. If distrust is excluded, and as the relationship continues, trust between the two parties evolves, and the nature of this trust will become more complex and multidimensional (Huang and Wilkinson, 2013). Another explanation for the business professionals’ preference for a trustworthy business consultant face may lie in the fact that communication problems are expected between professionals and consultants, because both are from different *thought worlds*, and professionals look for a consultant with whom they expect minimal communication problems (Sutter and Kieser, 2019).

Being not unattractive appears to be more important than fWHR for preference of business professionals for consultants. There may be a covariance between attractiveness and intelligence, and both are suggested to indicate “good genes,” but such covariance should be approached with care (Kleisner et al., 2014). Thus, we included the two characteristics attractiveness and intelligence as separate variables. The “good genes” argumentation would hold specifically for e-perceptions by women. However, we found no significant difference between men’s and women’s e-perceptions. Because we could not include perceived intelligence in the conjoint analysis, we could not test for any covariance.

Measuring fWHR based on photographs is not wholly reliable: if one measured fWHR based on different photographs of the same person, this would result in different fWHRs (Kramer, 2016). However, this does not affect our study. Here, we only used fWHR to compile profiles; thus, it is not about absolute fWHR but about relative fWHR. Further, we did not strive for an exact measure of the *de facto* fWHRs, but to see whether or not *perceived* fWHR has influence.

What did affect the study results is the fact that we conducted a field experiment and not a laboratory experiment. We used real photographs of real business consultants and asked real, highly educated business professionals to judge them in a zero-acquaintance situation. Because of the use of actual instead of manipulated photographs, there was variation among the photos that is not covered by the factors in the conjoint analysis. This is likely creating heterogeneity in the data, as reflected by the relatively high standard errors. However, this methodology has improved our study’s ecological validity.

Like Rule and Ambady (2008), who showed much more diversity in the responses to the faces than in the faces of CEO themselves, our results are remarkable, given that the 10 consultants who had to be ordered according to preference were so similar. They were all male, all around the same age, all white, all clean-faced (i.e., no glasses, no facial hair, etc.), all wearing the same kind of suit, and were presented against the same kind of background. The consultancy from which the business portraits were derived likely has implicit rules by which consultants are selected and presented, which affected the e-perceptions of these consultants (Adame and Bisel, 2019). Since we focused on fWHR, and since the behavioral effects that accompanied fWHR are especially shown for males, we included no business portraits of female consultants. This provoked some reactions from the respondents: we were asked why no females were involved. Remarkably, we received no such reactions concerning race. Apparently, it was more noticeable that the consultants were all male than that they were all white. Thus, it is worth investigating the first impression effect in the context of gender and race. Concerning gender, since female leaders are perceived differently to male leaders (Lauterbach and Weiner, 1996; Rule and Ambady, 2009), this likely also holds for female consultants.

Because we used real photographs of real business consultants, we had only 24 stimuli for the first survey, used to measure the estimated age, attractiveness, perceived intelligence, and perceived kindness of the consultants. Of course, more stimuli would have helped us to gain more statistical power. Including a lot of stimuli has, however, a pitfall as well. Since confronting respondents with a large number of stimuli involves a high cognitive cost for the respondent, lengthy questionnaires can cause exhaustion among respondents which might evoke a response bias (Morii et al., 2017; O’Donovan et al., 2020). This has a negative effect on the reliability of the data. Lengthy questionnaires with a lot of stimuli can also create a habituation effect (Harris, 1943), which reduces the response rate and weakens the statistical power (Morii et al., 2017). Considering the pros and cons of lengthy questionnaires with a lot of stimuli, we consider 24 stimuli to be an adequate amount, specifically since our stimuli were relatively homogeneous. In order to achieve a collection of stimuli that was as homogeneous as possible, we corrected for many variables that typically make photographs differ, such as glasses, facial hear, clothing, skin color, and gender. Furthermore, the photographs were all taken by the same photographer, and with the same camera, background and arrangement.

Based on the Generate Orthogonal Design procedure by PSS, we selected 10 cases out of the 24 stimuli and presented them by questionnaire to potential buyers of consultancy from our network, i.e., convenience sampling. One advantage of convenience sampling is that it is easy for respondents to express their reactions, and the study evoked some emotional responses. We received reactions that varied from “so much fun to do” to “I don’t judge purely based on faces.” The survey evoked a feeling of superficiality. But is this feeling legitimate? It has been suggested that the naïve inferences people make from facial appearance not only provides information about subjective preferences, but also about objective ones (Rule and Ambady, 2008). Even if people are provided with other information than just the information cues presented by a face—for instance, information of past behaviors—they will invest more in someone who looks trustworthy than in someone who looks untrustworthy (Rezlescu et al., 2012). As Adame and Bisel stated: “*Individuals cannot possibly make interpretations based on absolute information and are, therefore, constrained to select a fragment from which to interpret the whole.”* (Adame and Bisel, 2019, p. 24). The influences of psychological and physiological micro-foundations of choice behaviors will likely always be accompanied by feelings of superficiality or by perceptions that they influence others’ behaviors but not our own, specifically not in a professional context (Ariely, 2009).

### Limitations and Future Research

One study limitation is that we could not run the conjoint analysis the way we initially wanted. In an ideal situation, we could have included more factors and more levels for each factor in the orthogonal design. However, we had to omit one factor (perceived intelligence) and we had to dichotomize two factors instead of a polytomization (perceived (un)attractiveness and perceived kindness). Dichotomization is discussed in the literature (for an overview, see Butts and Ng, 2009), because it creates an ambiguous situation predominantly around the classification of items near the cutoff point. To deal with this pitfall of categorization, we asked all the 62 subjects that judged the consultants’ faces on estimated age, attractiveness, perceived intelligence and perceived kindness the same question in two ways (in an estimating way and a categorizing one), so that we could thoroughly find the best fit for every card in the orthogonal array. There is no problem of information loss owing to dichotomization, since the data on the perceptions of the faces was not used for data analysis, but only to be able to match the photographs to the cards in the orthogonal array such that the best fit was achieved.

Another limitation is that the study results (i.e., that business professionals prefer a small trustworthy face over a wide successful one) tells us nothing about the underlying motivations and adopted regulatory foci of business professionals expressing their preferences (Higgins, 1997). In other words, do business professionals want to maximize their gains when selecting a consultant (a promotion focus) or do they somehow seek to minimize loss (a prevention focus)? Thus, the theoretical contribution regarding the involved underlying mechanisms is limited, which is something future research can resolve.

Scholars studying fWHR noted that the lack of female stimuli is a limitation in the field (Geniole et al., 2015). Since this study rests upon the assumption of a, for males, well-established relationship between facial trait and behavioral construct, i.e., a relative wide face is supposed to communicate success and a relative small face is supposed to communicate trust, and this relationship is not yet established for females, we did not include female stimuli in our study. Since there are some studies that state that fWHR is not a sexually dimorphic trait (Lefevre et al., 2012; Özener, 2012), there is still a road to explore considering the relationship between female fWHR and behavior. It is likely that female fWHR can be linked to *other* behaviors than male fWHR (Arnocky et al., 2018). What those behaviors are and how they impact the perception of the other in the context of business mating is yet to be found out.

This study is skewed more toward e-perceptions than toward online impression management. In the field of business-to-business e-perceptions and online impression management, much remains to be discovered. One of these things to be discovered, and which we did not consider, is the digital communication types’ effects on e-perceptions (Murphy and Sashi, 2018). In this study, it became clear that profile pictures matter, not only in e-perceptions of the business professionals, but also regarding the likelihood of business mating. However, since relative face width is an implicit cue that is not deliberately changed to manage the impression formed by the other, it tells us nothing about the *de facto* success of impression management. Generally, the success of impression management is an under-researched domain (Stopfer et al., 2014).

It is possible that other facial structures, such as facial asymmetry, also influence this first impression, given attractiveness’ relative importance, as found in this study. The relative impacts of other facial structures and appearances remain for future research. Also, researchers should shed light on other micro-foundations of choice behaviors at work in a professional context; not only other facial or body metrics, but also the influences of hormones and mental states, which we already know do influence choice behaviors in a private context (e.g., Kosfeld et al., 2005; Adams et al., 2006; Durante et al., 2014; Durante and Laran, 2016; Nepomuceno et al., 2016). This exploratory study has shown that facial metrics do influence first impressions in the context of business mating, and has paved the way for further research along this track.

### Practical Implications and Conclusion

Does a *successful* face attract a business professional with whom one is not yet known? To the extent that a *successful* face is represented by a high fWHR, the answer was “not really.” Although in our data, one cluster of respondents was attracted to a *successful* face, we generally predominantly want business consultants to have a *not unattractive* face and to have a *trustworthy* face (i.e., a low fWHR).

E-perceptions of fWHR can be altered by how a person is photographed. A different head position impacts on the *perceived* fWHR. For instance, if the head is tilted upward or downward, the perceived fWHR is higher (Hehman et al., 2013b). Further, facial expressions do have an impact on perceived fWHR of a person in a photograph (Kramer, 2016). Specifically, a happy expression (i.e., a smiling face), positively affects perceived fWHR. Although one can affect perceptions of fWHR, one cannot change fWHR, except by undergoing plastic surgery. But in the digital world, it is possible to make a face wider or a smaller face by digitally manipulating an image.

E-perceptions of consultants’ business portraits are crucial, owing to the chance of being selected for a first interview. Consultants are often very aware of the importance of the impression they make on clients, and infuse their language with metaphors, images, and beliefs in order to impress them (Clark and Salaman, 1998; Alvesson, 2001; Nikolova et al., 2009). Nikolova et al. (2009) identified two impression management strategies for consultants: impressing via rhetoric by utilizing well-presented ideas, and impressing via tangible solutions by presenting empirically proven success stories. These are both behavioral strategies. In this study, we have revealed a third strategy based on the communication effects of the digital presentation of the self.

With this study we show businesses to think twice when and how they show profile photographs on their websites and on social media sites. These profile pictures influence the first impression potential business partners form, which in turn influences their preferences. So, if profile photographs are dominant elements on a firm’s website or on a social media website, which is common in businesses in high-knowledge service markets, it may be wise to test whether or not they provoke an approach-motivated intention.

The study results underline the importance of photographs as part of a professional communication strategy. The adage “a picture is worth a thousand words” exists for good reason, and emphasizes the suggestion that a successful communication strategy should be holistic and should have both verbal and visual elements. At the organizational level, it is known and widely accepted that visual representations outperform text (Kernbach et al., 2015). However, visual representations of corporate strategies imply a different communication strategy than pictures of individuals who belong to an organization. By presenting pictures of individuals on a corporate website, visitors to this website form impressions about these individuals, but they also form impressions about the firm and profession that the individual represents (Ewing et al., 2001). From this perspective, it may be wise to reassess the order in which the different profiles are presented on a corporate website. In practice, most firms currently use an alphabetical or a hierarchical profile order, when an order based on characteristics that create an approach tendency in a perceiver may well be better. We have addressed the power of individual photographs as part of a corporate communication strategy. The decision whether or not such power should be used is made at the organizational level, and it may involve ethical considerations concerning the persuasive elements of communication. Online impression management by the firm, if conducted correctly, is a financially wise investment (Schniederjans et al., 2013).

Besides the power of photographs as part of a firm-level communication strategy, one should also think about a communication strategy at the individual level. Since the rise of professional social media platforms such as LinkedIn, there is a stronger emphasis on the visibility and positioning of individual professionals. Broadcasting the self is now possible in ways that were not possible before (Krämer and Winter, 2008). Professionals mostly use LinkedIn, “*the largest professional matchmaker site in the world*” (van Dijck, 2013, p. 207), for self-promotion. They can use platforms such as LinkedIn strategically, as part of a personal branding strategy. Part of such a personal branding strategy is the photograph of oneself that one shares with one’s online network. The online environment offers a business professional the perfect setting to present the unique self, i.e., the cues that make one different to other individuals (Stopfer et al., 2014).

Whether at the corporate or at the individual level, contemporary online impression management demands picture-text alignment. When cues are everywhere, we may as well ‘help’ a perceiver by presenting matching cues. In his classic work on forming impressions, Asch (1946, p. 284) noted: “*There is an attempt to form an impression of the* entire *person. The subject can see the person only as a unit; he cannot form an impression of one-half or of one-quarter of the person.”* The context in which an impression is formed, and specifically the words used, influence the perception of the entire person. Asch (1946) already showed the impact of the word *warm* (compared to *cold*) on impression formation, but this impact is even stronger when the target is more distant, both in time and space (McCarthy and Skowronski, 2011). This may not only explain the dominance of the approach motivations in the *warm* honesty/humility dimension (see Table 5), even when a *successful* face is preferred over a *trustworthy* one, but also stresses the importance of using the ‘right’ words in online descriptions of business professionals, specifically in zero-acquaintance situations. When one wants to attract a business professional in the context of business mating, using words in the warm domain of honesty/humility may help.

This study also underlines the importance of being mindful of facial stereotyping, i.e., biased judgments based on facial characteristics. People’s reliance on facial cues may cause other relevant cues to be neglected, which may cause suboptimal outcomes. According to Olivola et al. (2014, p. 566), facial stereotyping affects our success: “*Our success and well-being, as individuals and societies, depend on our ability to make wise social decisions about important interpersonal matters, such as the leaders we select and the individuals we choose to trust. Nevertheless, our impressions of people are shaped by their facial appearances and, consequently, so too are these social decisions.”* On the other hand, we let ourselves be guided by facial stimuli, simply because we do. Two-month-old babies already pay more visual attention to attractive than unattractive faces (Langlois et al., 1987). So, while we cannot stop facial stereotyping, we can increase awareness of the phenomenon and of ways to minimize its effects. Future research is needed to develop counter-stereotype strategies concerning facial stereotyping, which will improve decision-making.

Every business success story has a beginning, and we have shed light on visual communication factors that may influence the success of such beginnings. In this initial phase, especially, physical appearance matters. A facial structure (e.g., fWHR) influences the judgments business professionals make of business consultants when they consider inviting them for a first interview. This first interview is crucial in the process of business mating and creating value together: “*while firms do not produce babies together, they do co-produce value for each other and for other firms in their networks through their interactions and adaptations over time”* (Wilkinson et al., 2005, p. 673). Having a sound corporate visual communication strategy will help to develop this process of co-producing value.

## Data Availability Statement

The raw data supporting the conclusions of this article will be made available by the authors, without undue reservation. The raw data of the survey can be found at https://data.4tu.nl/.

## Ethics Statement

Ethical review and approval was not required for the study on human participants in accordance with the local legislation and institutional requirements. The patients/participants provided their written informed consent to participate in this study.

## Author Contributions

EZ and JH developed the idea behind the research project together. EZ carried out the experiment and wrote the manuscript with support of JH. JH suggested the main analytical methods and supervised the research project. Both authors discussed the results and contributed to the final manuscript.

## Conflict of Interest

The authors declare that the research was conducted in the absence of any commercial or financial relationships that could be construed as a potential conflict of interest.

## Footnotes

1 The raw data of this survey are publicly available via data.4tu.nl
